# Propofol improved hypoxia‐impaired integrity of blood‐brain barrier via modulating the expression and phosphorylation of zonula occludens‐1

**DOI:** 10.1111/cns.13101

**Published:** 2019-01-24

**Authors:** Wei Chen, Xing‐Zhu Ju, Yan Lu, Xiao‐Wei Ding, Chang‐Hong Miao, Jia‐Wei Chen

**Affiliations:** ^1^ Department of Anesthesiology Fudan University Shanghai Cancer Center Shanghai China; ^2^ Department of Oncology, Shanghai Medical College Fudan University Shanghai China; ^3^ Department of Gynecologic Oncology Fudan University Shanghai Cancer Center Shanghai China

**Keywords:** blood‐brain barrier, hypoxia, mouse brain microvascular endothelial cells, propofol, zonula occludens‐1

## Abstract

**Aims:**

Hypoxia may damage blood‐brain barrier (BBB). The neuroprotective effect of propofol has been reported. We aimed to identify whether and how propofol improved hypoxia‐induced impairment of BBB integrity.

**Methods:**

Mouse brain microvascular endothelial cells (MBMECs) and astrocytes were cocultured to establish in vitro BBB model. The effects of hypoxia and propofol on BBB integrity were examined. Further, zonula occludens‐1 (ZO‐1) expression and phosphorylation, hypoxia‐inducible factor‐1α (HIF‐1α) and vascular endothelial growth factor (VEGF) expression, intracellular calcium concentration and Ca^2+^/calmodulin‐dependent protein kinase II (CAMKII) activation were measured.

**Results:**

Hypoxia‐impaired BBB integrity, which was protected by propofol. Hypoxia‐reduced ZO‐1 expression, while induced ZO‐1 phosphorylation. These effects were attenuated by propofol. The expression of HIF‐1α and VEGF was increased by hypoxia and was alleviated by propofol. The hypoxia‐mediated suppression of ZO‐1 and impaired BBB integrity was reversed by HIF‐α inhibitor and VEGF inhibitor. In addition, hypoxia increased the intracellular calcium concentration and induced the phosphorylation of CAMKII, which were mitigated by propofol. The hypoxia‐induced phosphorylation of ZO‐1 and impaired BBB integrity was ameliorated by calcium chelator and CAMKII inhibitor.

**Conclusion:**

Propofol could protect against hypoxia‐mediated impairment of BBB integrity. The underlying mechanisms may involve the expression and phosphorylation of ZO‐1.

## 1 INTRODUCTION

The blood‐brain barrier (BBB) is a highly selective semipermeable border that separates the circulating blood from the brain and extracellular fluid in the central nervous system (CNS). It restricts the diffusion of large or hydrophilic molecules, while allows the diffusion of hydrophobic and small polar molecules. This barrier is critical to maintain brain homeostasis, and the structural and functional integrity of the BBB appears to be dramatically altered by various noxious stimuli such as hypoxia, ischemia, and inflammation in the CNS, resulting in cerebral edema and neuron damage as well as brain dysfunction.^1^ In addition, neurological diseases, such as stroke, postoperative cognitive dysfunction, traumatic brain injury, and Alzheimer's disease, are associated with BBB dysfunction.^2^

Blood‐brain barrier is composed of endothelial cells of the capillary wall, astrocytes ensheathing the capillary, pericytes embedded in the capillary basement membrane, microglias, neurons, and noncellular component resulting from the extracellular matrix. Major BBB properties are possessed by the brain vascular endothelial cells. It was recognized that the proper function of the brain vascular endothelial cells is ensured by the presence of tight junction proteins, such as zonula occludens (ZOs), occludins, claudins, and junctional adhesion molecules (JAMs).^3^

Zonula occludens‐1 (ZO‐1) belongs to the family of ZOs and is proved to play an important role in keeping BBB integrity.^4^ The function of ZO‐1 may be modulated by its expression level and phosphorylation status. Multiple stimuli such as anesthesia and hypoxia may reduce the expression of ZO‐1, thus impairing the permeability of BBB in the in vitro and animal studies. Meanwhile, increased expression of ZO‐1 is found to be correlated with improved BBB integrity.^5^, ^6^, ^7^, ^8^, ^9^ Furthermore, it was reported that proinflammatory cytokines such as tumor necrosis factor‐α (TNF‐α) and interleukin‐6 (IL‐6) increased the phosphorylation of ZO‐1 and impaired BBB integrity in microvascular endothelium.^10^ A previous in vitro study carried out in porcine brain‐derived microvascular endothelial cells demonstrated that hypoxia may decrease ZO‐1 expression, while increase ZO‐1 phosphorylation.^11^ It was also showed that hypoxia may impair in vitro BBB integrity.^11^

Propofol is a widely used general anesthetic agent and has been proved to possess neuroprotective effects in hippocampal neurons and microglias which were exposed to TNF‐α, hypoxia, and Angiotensin II.^12^, ^13^, ^14^, ^15^ In addition, propofol has been shown to exert neuroprotective effects on the blood‐spinal cord barrier in rabbits after ischemia/reperfusion injury.^16^ More importantly, they reported that the protective effects of propofol were mediated by modulating the expression of tight junction protein occluding and claudin‐5.^16^ However, till to now, no data regarding the effects of propofol on the expression and phosphorylation of ZO‐1 is available.

In the present study, mouse brain microvascular endothelial cells (MBMECs) and mouse astrocytes were cocultured to establish in vitro BBB model. We examined the effects of hypoxia and propofol on ZO‐1 expression and phosphorylation as well as BBB integrity. More importantly, we aimed to clarify the underlying mechanisms. Although it was suggested that hypoxia‐induced ZO‐1 downregulation was mediated by vascular endothelial growth factor (VEGF),^11^ the mechanism for hypoxia‐induced ZO‐1 phosphorylation has not been examined. Since hypoxia is known to be correlated with calcium overload and calcium signaling pathway, these factors were investigated in this study.

## 2 METHODS

### 2.1 Experimental design

The study is composed of two parts. Firstly, MBMECs and mouse astrocytes were cocultured to establish in vitro BBB model, which was exposed to normoxia condition (95% air, 5% CO_2_) or hypoxia condition (5% O_2_, 5% CO_2_, 90% humidity) for 3 hours. The hypoxia condition was achieved by using hypoxia chamber (Ruskinn Technologies, Leeds, UK). To examine the effect of propofol, in vitro BBB model was pretreated with different concentrations of propofol (5, 10, 25, 50, 100, 200 μmol/L) or its solvent 0.1% dimethyl sulfoxide (DMSO) for 1 hour, followed by hypoxia condition treatment for 3 hours. BBB integrity was examined by measuring trans‐endothelial electrical resistance (TEER). Thus, we planned to identify the concentration of propofol that could protect hypoxia‐impaired BBB integrity. Secondly, MBMECs were exposed to normoxia condition, hypoxia condition, or pretreated with propofol followed by hypoxia condition. The effects of hypoxia and propofol on ZO‐1 expression and phosphorylation were examined, and the underlying mechanisms, including hypoxia‐inducible factor‐1α (HIF‐1α)/vascular endothelial growth factor (VEGF) and calcium/calmodulin‐dependent protein kinase II (CAMKII) were investigated. To confirm the role of these factors, the effects of specific inhibitors to HIF‐1α (KC7F2), VEGF (CBO‐P11), CAMKII (KN93), and calcium chelator (BAPTA) were examined.

### 2.2 Cell culture

Mouse brain microvascular endothelial cells were purchased from Shanghai WeiKe Company (Shanghai, China) and cultured in endothelial basal medium (Sigma‐Aldrich, Shanghai, China) containing vascular endothelial growth factor, insulin‐like growth factor, 10% fetal bovine serum (FBS), 100 units/mL penicillin and 100 μg/mL streptomycin. Media were replenished every 2‐3 days. On reaching 80%‐90% confluency, cells were subcultured. The 6th passage of cells was used in this study.

Mouse astrocytes were obtained from Shanghai WeiKe Company (Shanghai, China), and cultured in Dulbecco's modified eagle medium (DMEM) (Sigma‐Aldrich, Shanghai, China) with 10% FBS, 100 units/mL penicillin and 100 μg/mL streptomycin. Media were replaced every 2‐3 days, and cells were subcultured when reaching 80%‐90% confluency. The 3rd passage of cells was used in the present study.

### 2.3 Establishment of in vitro BBB model

In vitro BBB model was built by coculturing MBMECs and mouse astrocytes on opposing sides of 24‐well cell culture inserts with 3.0 μm pores (Sigma‐Aldrich, Shanghai, China). The inserts were coated with poly‐L‐lysine on the underside and type I collagen on the topside. Mouse astrocytes were seeded at a density of 15 000 cells/cm^2^ on the underside, and MBMECs were seeded at a density of 25 000 cells/cm^2^ on the topside. Subsequently, inserts were cultured in endothelial basal medium containing 10% FBS. Media were replenished every 2‐3 days, and TEER was measured every day to assess the integrity of in vitro BBB model. In this study, in vitro BBB model was ready for experiments after 6 days coculturing, and thereafter, all experimental procedures were performed in serum‐free media.

### 2.4 Measurement of TEER

Trans‐endothelial electrical resistance was measured by using epithelial voltmeter (World Precision Instruments, FL, USA). In brief, after respective treatment, media were aspirated from the upper chamber and cell culture inserts were transferred to a new well and washed with phenol‐red free DMEM. TEER was analyzed with an epithelial voltmeter (World Precision Instruments, FL, USA), and the values were expressed as Ω*cm^2^. According to previous findings (6, 9, 11), when the TEER value reached over 300 Ω*cm^2^, the BBB model was considered validly established.

### 2.5 Protein preparation and analysis by Western blot

After treatment, MBMECs were collected and homogenized in RIPA lysis buffer containing 50 mmol/L Tris, 150 mmol/L NaCl, 1% Triton X‐100, 1% sodium deoxycholate, 2 mmol/L EDTA, protease inhibitor, and phosphatase inhibitor. The lysate was centrifuged for 20 min at 10 000 g at 4°C, and the protein content was determined by BCA assay (Sigma‐Aldrich, Shanghai, China). Same amount of proteins (about 60 μg) was loaded per lane, separated by sodium dodecyl sulfate polyacrylamide gel electrophoresis, and transferred to polyvinylidene difluoride membranes. After being incubated in 5% nonfat dry milk solution for 1 hour, membranes were probed with specific primary antibody diluted in 5% nonfat milk at 4°C for overnight. Primary antibodies were obtained from Santa Cruz Biotechnology (Santa Cruz, CA, USA) and included antibody against ZO‐1, phosphorylated ZO‐1, HIF‐1α, VEGF, CAMKII, phosphorylated CAMKII, and GAPDH. Then, the membranes were washed with tris‐buffered saline and incubated with corresponding secondary antibody (Santa Cruz, CA, USA) at room temperature for 1 hour. After being washed, protein bands were detected with Amersham ECL plus Western blotting detection reagent (Santa Cruz, CA, USA), and densitometry analysis was performed with Gel‐IT analysis software.

### 2.6 Measurement of intracellular free calcium concentration

Intracellular free calcium concentration was detected by the fluorescent dye Fluo‐3 AM (Beyotime biotechnology, Shanghai, China). In brief, after respective treatment, cells were harvested by scraping, washed with phosphate buffer saline, and suspended in 5 μmol/L Fluo‐3 AM for 45 min in the dark room. Fluorescence was monitored by flow cytometry at 528 nm (excitation: 490‐500 nm). Data were expressed as fluorescence intensity.

### 2.7 Statistical analysis

Data were demonstrated as mean ±standard deviation. Sample size (n) represents the times of repeated experiments which were performed with different cell cultures. Statistical comparisons were made by paired Student's *t* test, Student Newman‐Keuls test (*q* test), one‐way ANOVA followed by Tukey's post hoc test. All statistical analyses were performed with SPSS software 10.0, and a *P* value less than 0.05 was considered statistically significant.

## 3 RESULTS

### 3.1 The effects of Hypoxia and Propofol on BBB integrity in the in vitro model

**Figure 1:**
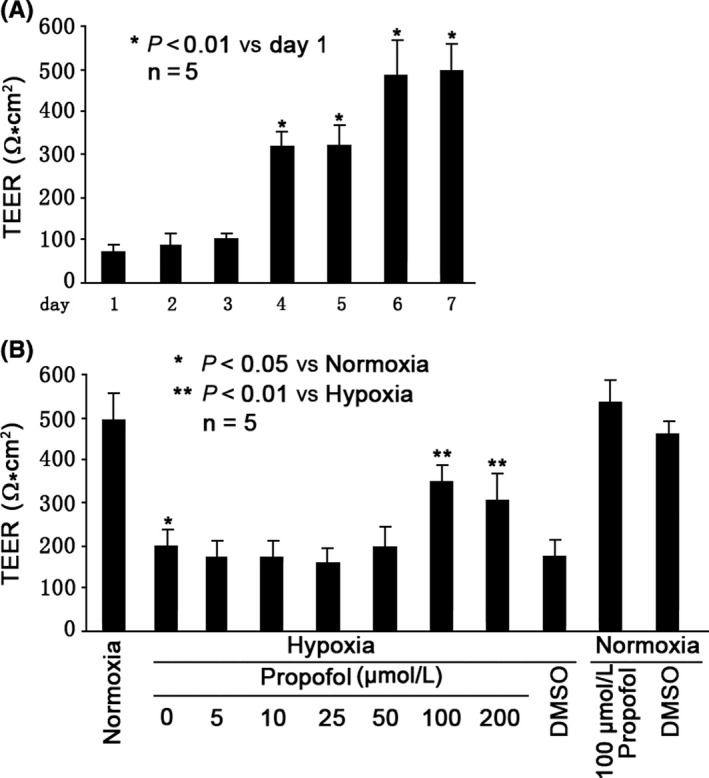
Propofol protected hypoxia‐impaired BBB integrity. A, Evaluation of in vitro BBB integrity by measuring TEER over the course of 7 days coculturing of MBMECs and mouse astrocytes at normoxia condition (95% air, 5% CO_2_). Data showed TEER peak point at day 6, suggesting the construction of in vitro BBB model. B, Hypoxia (5% O_2_, 5% CO_2_, 90% humidity) impaired BBB integrity, which was protected by 100 μmol/L propofol pretreatment. TEER values were expressed as Ω*cm^2^, presented as mean ±standard deviation, and summarized from five separate experiments. Statistical comparisons were made by paired Student's *t* test, one‐way ANOVA followed by Tukey's post hoc test (Student's Newman‐Keuls test)

The integrity of in vitro BBB model was examined by measuring TEER after coculturing of MBMECs and mouse astrocytes at normoxia condition for 1, 2, 3, 4, 5, 6, and 7 days, respectively. As shown in Figure 1A, TEER reached 300Ω*cm^2^after 4 days coculturing of endothelial cells and astrocytes, suggesting the successful establishment of in vitro BBB model. And TEER peaked after 6 days coculturing of endothelial cells and astrocytes, suggesting the optimal condition for in vitro BBB model. Further, we demonstrated that the integrity of in vitro BBB model was impaired by hypoxia condition treatment for 3 hours (*P* < 0.05 vs normoxia condition, Figure 1B). In addition, we reported pretreatment of the in vitro BBB model with propofol (5, 10, 25, 50, 100, and 200 μmol/L) for 1 hour could protect the hypoxia‐impaired BBB integrity in a concentration‐dependent manner, and the maximal effect was observed at 100 μmol/L (*P* < 0.01 vs hypoxia condition, Figure 1B). Please be noticed that the solvent for propofol, 0.1% DMSO, had no such effect, and please also be noticed that 100μΜ propofol or 0.1% DMSO alone had no effect on BBB integrity under normoxia condition (Figure 1B).

### 3.2 The effects of Hypoxia and Propofol on ZO‐1 expression and Phosphorylation in MBMECs

**Figure 2:**
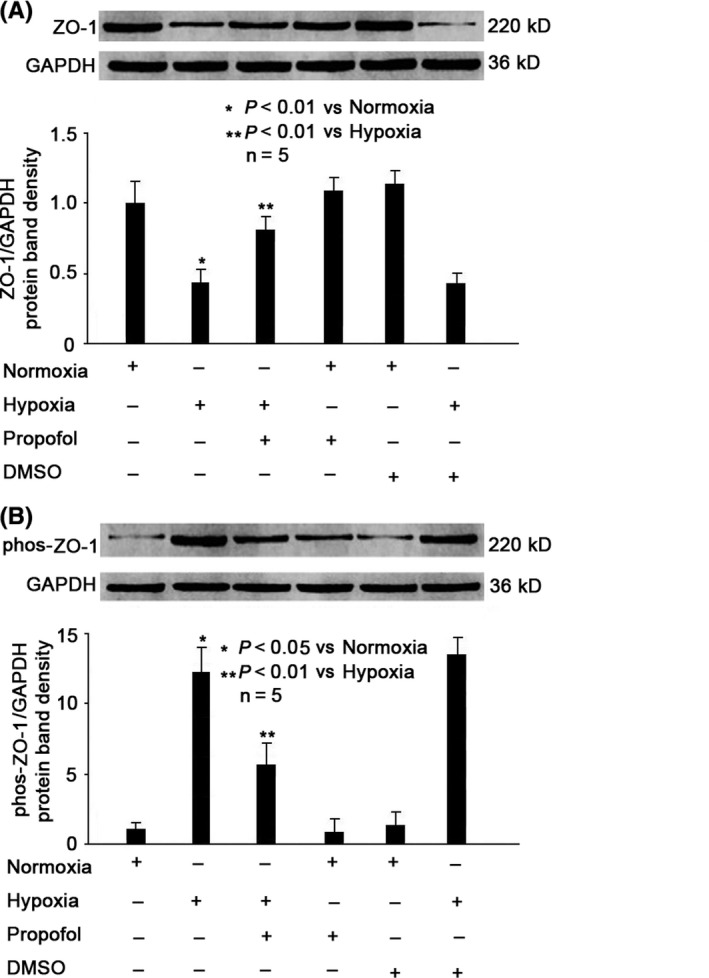
Propofol reversed hypoxia‐mediated ZO‐1 expression and phosphorylation. A, Hypoxia‐reduced protein expression of ZO‐1, which was increased by 100 μmol/L propofol. The upper panel was a representative experiment, and the lower panel was the summary of densitometric data from five separate experiments. GAPDH served as loading control. Data were expressed as normalized ratio of protein band density of ZO‐1 against GAPDH and were presented as mean ±standard deviation. B, Hypoxia‐induced phosphorylation of ZO‐1, which was inhibited by 100 μmol/L propofol. The upper panel was a representative experiment, and the lower panel was the summary of densitometric data from five separate experiments. GAPDH served as loading control. Data were expressed as normalized ratio of protein band density of phosphorylated ZO‐1 against GAPDH and were presented as mean ±standard deviation. Statistical comparisons were made by paired Student's *t* test, one‐way ANOVA followed by Tukey's post hoc test (Student's Newman‐Keuls test)

As shown in Figure 2, we found in MBMECs that compared with normoxia condition, hypoxia could greatly reduce the expression of ZO‐1 (*P* < 0.01, Figure 2A), while increase the phosphorylation of ZO‐1 (*P* < 0.05, Figure 2B). In addition, we demonstrated that the hypoxia‐modulated expression and phosphorylation of ZO‐1 were mitigated by 100 μmol/L propofol pretreatment (*P* < 0.01 vs hypoxia condition, Figure 2A,B), but were not affected by 0.1% DMSO pretreatment. Also, we found that 100 μmol/L propofol or 0.1% DMSO alone had no effect on ZO‐1 expression and phosphorylation under normoxia condition (Figure 2A and B).

### 3.3 Role of HIF‐1α and VEGF in Hypoxia‐ and Propofol‐modulated ZO‐1 expression in MBMECs

**Figure 3:**
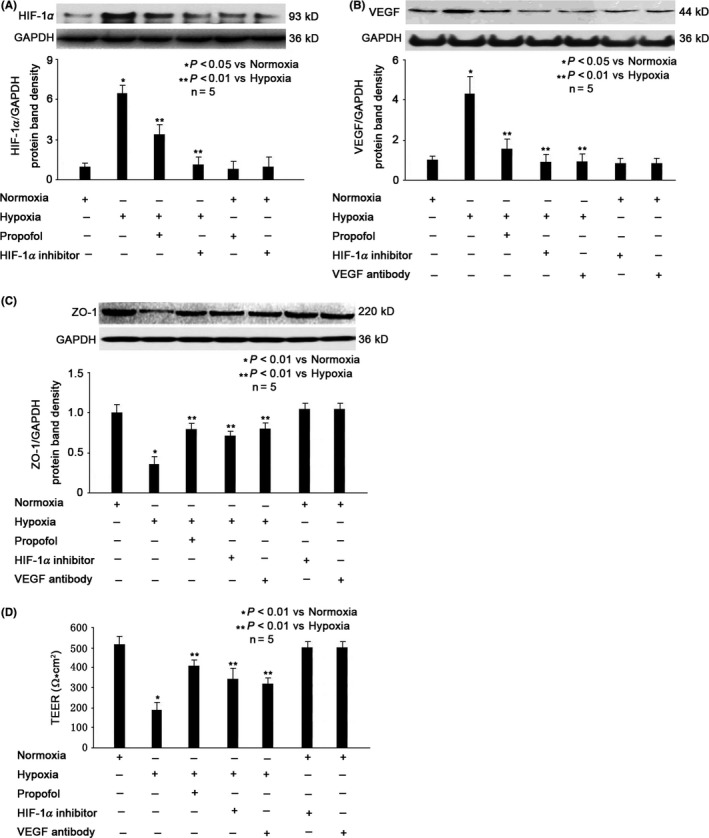
Role of HIF1α/VEGF in propofol‐ and hypoxia‐modulated ZO‐1 expression. A, Hypoxia increased HIF1α level, which was attenuated by 100 μmol/L propofol. The upper panel was a representative experiment and the lower panel was the summary of densitometric data from five separate experiments. GAPDH served as loading control. Data were expressed as normalized ratio of protein band density of HIF1α against GAPDH and were presented as mean ±standard deviation. B, Hypoxia‐reduced VEGF level, which was increased by 100 μmol/L propofol. The upper panel was a representative experiment, and the lower panel was the summary of densitometric data from five separate experiments. GAPDH served as loading control. Data were expressed as normalized ratio of protein band density of VEGF against GAPDH and were presented as mean ±standard deviation. C, Hypoxia‐reduced ZO‐1 expression was reversed by propofol, HIF1α inhibitor and VEGF inhibitor. The upper panel was a representative experiment, and the lower panel was the summary of densitometric data from five separate experiments. GAPDH served as loading control. Data were expressed as normalized ratio of protein band density of ZO‐1 against GAPDH and were presented as mean ±standard deviation. D, Hypoxia‐reduced BBB integrity was reversed by propofol, HIF1α inhibitor and VEGF inhibitor. TEER values were expressed as Ω*cm^2^, presented as mean ±standard deviation, and summarized from five separate experiments. Statistical comparisons were made by paired Student's *t* test, one‐way ANOVA followed by Tukey's post hoc test (Student's Newman‐Keuls test)

We showed that hypoxia induced the expression of HIF‐1α and VEGF (*P* < 0.05 vs normoxia, Figure 3A,B), which was attenuated by 100μΜ propofol pretreatment (*P* < 0.01 vs hypoxia, Figure 3A,B). To confirm the role of HIF‐1α, cells were pretreated with 20 μmol/L KC7F2 (a selective HIF‐1α translation inhibitor) for 1 hour, followed by hypoxia condition treatment for 3 hours. Our data proved that KC7F2 could ameliorate hypoxia‐modulated expression of HIF‐1α (*P* < 0.01 vs hypoxia, Figure 3A), VEGF (*P* < 0.01 vs hypoxia, Figure 3B), and ZO‐1 (*P* < 0.01 vs hypoxia, Figure 3C). Consistently, our data indicated that KC7F2 could protect hypoxia‐impaired BBB integrity, and the effect is similar to that of propofol (*P* < 0.01 vs hypoxia, Figure 3D). To confirm the role of VEGF, cells were pretreated with 10 μmol/L CBO‐P11 (a monoclone antibody against VEGF) for 1 hour, followed by hypoxia condition treatment for 3 hours. We found that CBO‐P11 could inhibit hypoxia‐induced expression of VEGF (*P* < 0.01 vs hypoxia, Figure 3B) and ZO‐1 (*P* < 0.01 vs hypoxia, Figure 3C) and could protect hypoxia‐impaired BBB integrity (*P* < 0.01 vs hypoxia, Figure 3D). We also indicated that the effect of CBO‐P11 is comparable to that of propofol (Figure 3B‐D).

### 3.4 Role of calcium and CAMKII in Hypoxia‐ and Propofol‐modulated ZO‐1 Phosphorylation in MBMECs

**Figure 4:**
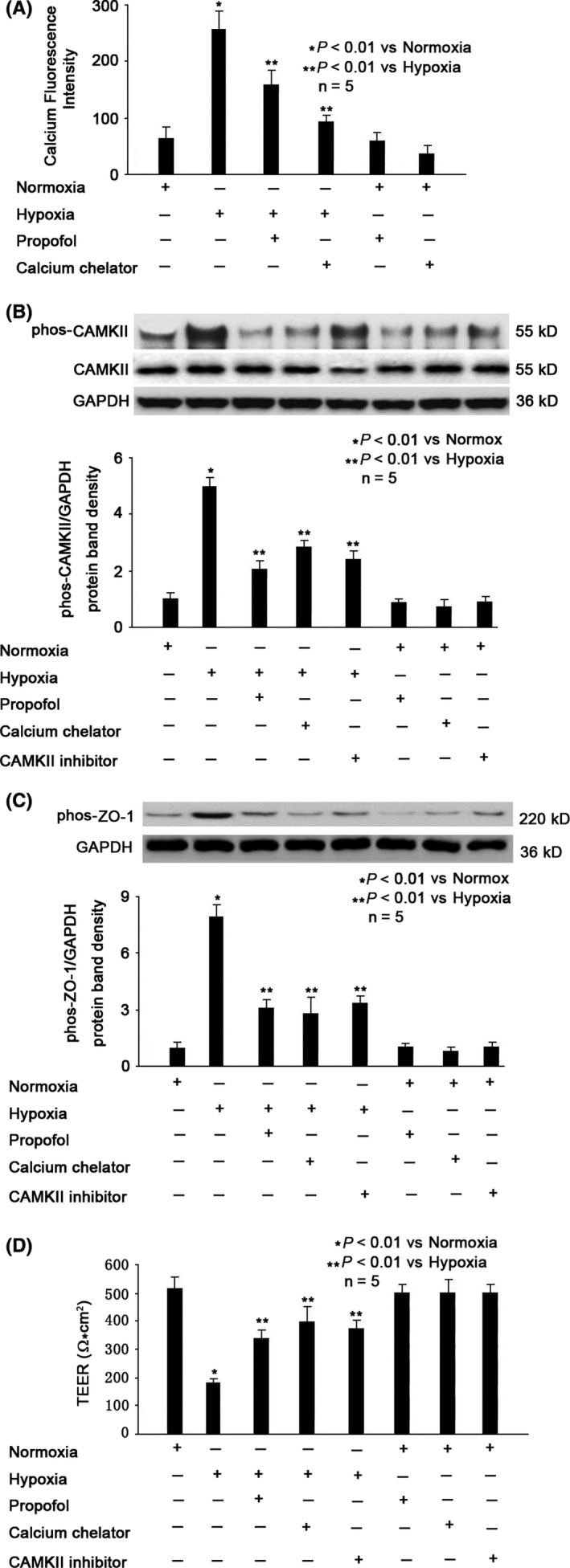
Role of calcium/CAMKII in propofol‐ and hypoxia‐modulated ZO‐1 phosphorylation. A, Hypoxia‐induced intracellular calcium concentration, which was attenuated by propofol and calcium chelator. Data were expressed as fluorescence intensity, presented as mean ±standard deviation, and summarized from five separate experiments. B, Hypoxia‐induced CAMKII phosphorylation, which was attenuated by propofol, calcium chelator and CAMKII inhibitor. The upper panel was a representative experiment, and the lower panel was the summary of densitometric data from five separate experiments. GAPDH served as loading control. Data were expressed as normalized ratio of protein band density of phosphorylated CAMKII against CAMKII, which was normalized with GAPDH, and were presented as mean ±standard deviation. C, Hypoxia‐induced phosphorylation of ZO‐1, which was inhibited by propofol, calcium chelator and CAMKII inhibitor. The upper panel was a representative experiment, and the lower panel was the summary of densitometric data from five separate experiments. GAPDH served as loading control. Data were expressed as normalized ratio of protein band density of phosphorylated ZO‐1 against GAPDH and were presented as mean ±standard deviation. D, Hypoxia‐reduced BBB integrity was reversed by propofol, calcium chelator, and CAMKII inhibitor. TEER values were expressed as Ω*cm^2^, presented as mean ±standard deviation, and summarized from five separate experiments. Statistical comparisons were made by paired Student's *t* test, one‐way ANOVA followed by Tukey's post hoc test (Student's Newman‐Keuls test)

As shown in Figure 4A, hypoxia significantly increased intracellular calcium concentration (*P* < 0.01 vs normoxia, Figure 4A), which was reduced by 100 μmol/L propofol pretreatment (*P* < 0.01 vs hypoxia, Figure 4A). Consistently, we showed that hypoxia caused phosphorylation of CAMKII (*P* < 0.01 vs normoxia, Figure 4B), which was mitigated by 100 μmol/L propofol pretreatment (*P* < 0.01 vs hypoxia, Figure 4B). To confirm the role of intracellular calcium, we pretreated cells with 100 μmol/L BAPTA (calcium chelator) for 1 hour, followed by hypoxia treatment for 3 hours. We demonstrated that BAPTA could inhibit hypoxia‐induced intracellular calcium overload (*P* < 0.01 vs hypoxia, Figure 4A), decrease phosphorylation of CAMKII (*P* < 0.01 vs hypoxia, Figure 4B) and ZO‐1 (*P* < 0.01 vs hypoxia, Figure 4C), and protect hypoxia‐impaired BBB integrity (*P* < 0.01 vs hypoxia, Figure 4D). The effect of BAPTA is similar to that of propofol (Figure 4A‐D). To confirm the role of CAMKII, we pretreated cells with 100 μmol/L KN93 (CAMKII inhibitor) for 1 hour, followed by hypoxia treatment for 3 hours. We demonstrated that KN93 could inhibit hypoxia‐modulated phosphorylation of CAMKII (*P* < 0.01 vs hypoxia, Figure 4B) and ZO‐1 (*P* < 0.01 vs hypoxia, Figure 4C), protect hypoxia‐impaired BBB integrity (*P* < 0.01 vs hypoxia, Figure 4D) and the effect of KN93 is similar to that of propofol (Figure 4B‐D).

## 4 DISCUSSION

### 4.1 The effects of propofol on hypoxia‐impaired BBB integrity

Hypoxia, referring to the oxygen demand of tissues is not met, is present in many pathological states including stroke, and it is a major risk factor for intraoperative brain injury, especially in elderly patients and in patients with restricted blood supply to the brain. It serves as an initial trigger for pathophysiological changes at the BBB, and causes damage of the CNS. A large number of in vivo and in vitro studies have demonstrated that hypoxia is a major stress factor that induces BBB disruption, leading to altered distribution of water and ions, inflammatory events and oxidative stress, edema formation, infiltration of peripheral immune cells and leakage of blood proteins into the brain.^17^, ^18^, ^19^ Further, accumulating evidence supports the role of hypoxia as one of the major factors leading to BBB dysfunction and a variety of CNS diseases, such as stroke, cognitive dysfunction, and dementia.^20^, ^21^ Consistently, in the current study, we examined the effect of hypoxia in an in vitro model and indicated that 3 hours hypoxia treatment significantly impaired BBB integrity. However, recent in vitro and animal studies reported that hypoxia may enhance BBB integrity.^4^ It should be noted that the hypoxia condition in those studies refers to mild hypoxia preconditioning (10% O_2_) or chronic mild hypoxia (8%‐10% O_2_, 2‐7 weeks), which is different from the hypoxia condition (5% O_2,_3 hours) applied in this study.

The neuroprotective effects of propofol are of great interests. Increasing evidence has supported potential neuroprotective efficacy in in vitro studies, animal studies, and clinical trials.^12^, ^13^, ^14^, ^15^, ^16^, ^22^, ^23^, ^24^, ^25^ The neuroprotective effects of propofol may be carried out through multiple mediators, among which BBB is one major target. It was reported in animal models that propofol may alleviate hypoxia‐impaired BBB integrity, thus protecting hypoxia‐induced cerebral edema and brain injury in rats.^22^, ^26^, ^27^ In the present study, we also reported that propofol may protect hypoxia‐impaired BBB integrity in the in vitro model. However, it is noted that the neuroprotective effects of propofol could be carried out through targeting other mediators, such as the apoptosis of hippocampal neurons.^15^

### 4.2 The involvement of ZO‐1 in hypoxia‐ and propofol‐regulated BBB integrity

The mechanisms of hypoxia‐impaired BBB integrity have been studied intensively, and increasing evidence has pointed to brain vascular endothelial cells and tight junction proteins. It has been shown that hypoxia could modulate the protein expression level and subcellular redistribution of ZOs, occludins, and claudins.^11^, ^28^, ^29^ Recently, more and more researches focused on the role of ZO‐1, which is essential to the proper assembly of interendothelial junction complexes that control BBB integrity. It was shown that hydralazine‐induced hypoxia may increase BBB permeability through decreasing ZO‐1 expression without affecting the expression of occludins and claudins in the in vitro BBB model.^9^, ^30^ Animal study also revealed that oxygen/glucose deprivation‐induced hypoxia may decrease ZO‐1 expression and impair BBB integrity in rats,^17^ while chronic mild hypoxia (10% O_2_, 7 weeks) may enhance BBB integrity by increasing ZO‐1 expression in mice.^4^ The unanimous change of ZO‐1 and BBB integrity strongly implied the key role of ZO‐1 in maintaining normal BBB structure and function. Beside ZO‐1 expression, the BBB integrity is also affected by the phosphorylation status of ZO‐1, because phosphorylated ZO‐1 may lose its normal function. It was reported that TNF‐α could reduce ZO‐1 expression, while enhance ZO‐1 phosphorylation in human brain microvascular endothelial cells, thus potentially affecting BBB permeability.^10^ Animal study also indicated that in dystrophic mice brains, decreased ZO‐1 expression, and increased ZO‐1 phosphorylation were correlated with impaired BBB integrity.^31^ In the present study, we reported that hypoxia may reduce ZO‐1 expression while increase ZO‐1 phosphorylation, leading to BBB integrity impairment.

Numerous studies indicated that the neuroprotective effect of propofol against BBB impairment could be mediated through multiple pathways. We found in an ongoing in vitro study that propofol could modulate TNF‐α‐induced matrix metalloprotein‐9 (MMP‐9) expression and activation, thus alleviating TNF‐α‐induced extracellular matrix breakdown and basal membrane damage (data not shown). Consistently, propofol was found to protect the BBB by decreasing MMP‐9 expression and improve the neurobehavioral outcome in rats.^26^ Others also reported that propofol could reverse ischemia‐downregulated expression of claudins and occludins, thus exerting protective effect on BBB in rabbits.^16^ In this study, we demonstrated that the neuroprotective effect of propofol against hypoxia‐impaired BBB was correlated with increased ZO‐1 expression and decreased ZO‐1 phosphorylation. Nevertheless, we could not completely rule out the potential effects of propofol on other tight junction proteins, such as claudins and occludins.

### 4.3 The role of HIF‐1α/VEGF pathway and calcium/CAMKII pathway in hypoxia‐ and propofol‐regulated BBB integrity

Previous studies have indicated that HIF‐1α is considered as a master regulator of the hypoxic response. It is composed of an oxygen‐sensitive α subunit and a constitutively expressed β subunit. Under normoxia situation, the HIF‐1α is constitutively transcribed but constantly targeted for proteasomal degradation through a cascade of hydroxylation, ubiquitination, and degradation by the proteasome. While under hypoxia condition, HIF‐1α is stabilized, translocates to the nucleus and dimerizes with HIF1β to form a functional HIF‐1 transcription factor, subsequently inducing the expression of target genes, such as VEGF, which is a strong inducer of BBB permeability by reducing ZO‐1 expression and is closely related to hypoxic‐ischemic brain injury.^11^, ^18^, ^31^, ^32^, ^33^, ^34^ Recently, the mechanism for high glucose‐induced BBB disruption was examined in MBMECs, and the pivotal role of HIF‐1α/VEGF/ZO‐1 was indicated.^35^

An in vitro study suggested that propopol may protect hypoxia‐impaired BBB permeability via modulating HIF‐1α expression.^36^ The effect of propofol against hypoxia‐induced HIF1α expression was also identified in prostate cancer cells^37^ and alveolar epithelial cells.^38^ It was shown that propofol downregulated HIF‐1α expression, leading to reduced VEGF expression in prostate cancer cells.^39^ In addition, animal studies proved that propofol exerts protective effects against ischemia‐induced BBB and liver damage, and HIF‐1α played a key role.^22^, ^40^ Consistently, in this in vitro study we indicated that hypoxia may induce HIF‐1α expression, which led to increased VEGF expression and reduced ZO‐1 expression, finally damaging BBB integrity. More importantly, by examining the effect of inhibitors to HIF‐1α and VEGF, our data clearly indicated the protective effect of propofol, and strongly implied that the protective effect was mediated through HIF‐1α/VEGF/ZO‐1 pathway.

As discussed earlier, ZO‐1 phosphorylation was correlated with BBB integrity. It was reported proinflammatory cytokines TNF‐α and IL‐6 increased ZO‐1 phosphorylation in human brain microvascular endothelial cells, but the mechanisms for ZO‐1 phosphorylation were not examined.^10^ In another study, it was demonstrated that ZO‐1 phosphorylation was correlated with BBB opening in the brains of dystrophic mice. Interestingly, the mechanisms for ZO‐1 phosphorylation were not examined as well.^31^ In a recent study performed in bovine brain endothelial cell line, hypoxic stress was found to enhance intracellular calcium overload.^41^ It was also reported that hypoxia‐induced calcium/CAMKII in rat brain capillary endothelial cells.^42^ The calcium overload and calcium signaling pathway were also found to be involved in hypoxia‐induced malfunction in other cells, such as pulmonary artery smooth muscles^43^ and hippocampal neurons.^44^ We demonstrated that hypoxia caused intracellular calcium overload and activated CAMKII, leading to ZO‐1 phosphorylation. We also found that hypoxia‐induced ZO‐1 phosphorylation was attenuated by calcium chelator and CAMKII inhibitor, implying the key role of calcium/CAMKII pathway.

The effects of propofol on calcium signaling pathway have been widely studied. Propofol has been proved to alleviate hypoxia‐induced calcium overload and CAMKII activation in hippocampal neurons^13^, ^44^ and microglias.^12^ In addition, propofol has been shown to protect inflammation cytokine‐induced injury in hippocampal neurons^14^ and microglias,^45^ via modulating calcium/CAMKII pathway. Propofol could also reduce intracellular calcium concentration and CAMKII activity in pancreatic cancer cells.^39^ We demonstrated in this study that propofol may inhibit hypoxia‐induced intracellular calcium accumulation and CAMKII activation as well as ZO‐1 phosphorylation, implying the protective effect was mediated through calcium/CAMKII/ZO‐1 pathway.

### 4.4 Limitations

Although we think this is a relatively complete study, we realize that limitations still exist. Firstly, we only examined the effects of hypoxia and propofol on BBB in the in vitro model. Our findings should be confirmed in the in vivo animal study, in which the effects of hypoxia and propofol on ZO‐1 expression, ZO‐1 phosphorylation, BBB integrity, and brain cognitive function need to be examined. Secondly, in the study we focused on ZO‐1, while, we could not rule out the involvement of other tight junction proteins. Further studies employing ZO‐1 over‐expression and ZO‐1 silencing technique may dissolve this issue. Thirdly, in this study, we suggested the intracellular calcium overload as a potential mechanism for ZO‐1 phosphorylation, but we did not investigate the source of calcium. Further experiments were needed to clarify whether calcium is from extracellular environment or from endoplasmic reticulum. Lastly, in this study, we only proved the correlation between CAMKII and ZO‐1 phosphorylation. However, we could not tell whether CAMKII directly caused ZO‐1 phosphorylation or it activated other kinases, which then caused ZO‐1 phosphorylation.

## 5 CONCLUSIONS

In this study, we demonstrated that propofol may protect hypoxia‐impaired BBB integrity in the in vitro model. Further, we focused on the underlying mechanisms and our data implied the involvement of ZO‐1 expression and phosphorylation. More importantly, we indicated that the hypoxia‐ and propofol‐regulated expression of ZO‐1 is mediated through HIF‐1α/VEGF pathway, and that the hypoxia‐ and propofol‐regulated phosphorylation of ZO‐1 is mediated through calcium/CAMKII pathway.

## CONFLICT OF INTEREST

The authors declare no conflict of interest.
